# Using Lamin B1 mRNA for the early diagnosis of hepatocellular carcinoma: a cross-sectional diagnostic accuracy study

**DOI:** 10.12688/f1000research.14795.1

**Published:** 2018-08-24

**Authors:** Amani M. Abdelghany, Nasser Sadek Rezk, Mona Mostafa Osman, Amira I. Hamid, Ashraf Mohammad Al-Breedy, Hoda A. Abdelsattar

**Affiliations:** 1 Department of Clinical Pathology, Faculty of Medicine, Ain Shams University, Cairo, Egypt; 2 Department of Tropical Medicine and Infectious Diseases, Faculty of Medicine, Ain Shams University, Cairo, Egypt

**Keywords:** Hepatocellular carcinoma, lamin B1, AFP, RT-qPCR

## Abstract

**Background** **:**  Hepatocellular carcinoma (HCC) is vital medical issue in Egypt. It accounts for 70.48% of all liver tumors among Egyptians. The aim of this study was to determine the diagnostic role of plasma levels of mRNA of lamin B1 by RT-qPCR as an early marker of HCC.

**Methods:** This study was conducted at the Clinical Pathology Department in collaboration with the Department of Tropical Medicine and Infectious Diseases at Ain Shams University Hospitals. It included 30 patients with primary HCC and viral cirrhosis (all were hepatitis C virus-positive) (Group I), in addition to 10 patients with chronic liver diseases (Group II) and 10 healthy age- and sex-matched subjects (Group III). Group I was further classified according to the Barcelona-Clinic Liver Cancer Staging System. Serum α-fetoprotein (AFP) chemiluminescent-immunoassays and RT-qPCR analysis of plasma lamin B1 mRNA levels were performed for all participants.

**Results:** AFP and lamin B1 significantly elevated in patients with HCC compared to those in the other studied groups. AFP and lamin B1 status could discriminate group I from group II and III. A significant increase was found among the three Barcelona stages with regards to AFP and lamin B1 levels. A significant decrease was found between group II and stage 0, A and B with regards to AFP and lamin B1. Lamin B1 and AFP could both differentiate HCC patients with one tumor nodule (T1) from those with two or more tumor nodules (T2&Tm), as well as between those with tumor sizes >3 cm and ≤3 cm.

**Conclusion: **Measurement of lamin B1 mRNA is recommended in patients with chronic liver disease with normal serum AFP, especially in known cirrhotic patients that deteriorate rapidly without any apparent etiology.

## Introduction

Hepatocellular carcinoma (HCC) is the most common primary malignancy of hepatocytes, representing the fifth most common cancer, worldwide ^1^. HCC is the third-leading cause of cancer-related deaths worldwide, accounting for approximately 1 million deaths annually ^2^. Infection with hepatitis C virus (HCV) and/or hepatitis B virus contributes to >60% of HCC cases. HCC is a large problem in low- and middle-income countries and regions ^3^. In Egypt, HCC accounts for 4.7% of all liver diseases. The relative frequency of all liver cancers in Egypt reached 7.3% in 2003 with 95% of them are HCC cases ^4^.

The nuclear lamina is a proteinaceous meshwork found below the inner nuclear membrane, composing of intermediate filament proteins: lamin A, lamin C and lamins B1 and B2. Lamins are essential for a number of cellular functions, including nuclear stability, chromatin structure and gene expressions ^5^. B-type lamins are widely expressed in most cell types, including embryonic stem cells ^6^. Lamin B1 was found to be essential for nuclear integrity, cell survival and normal development ^7^. However, neither cell proliferation nor skin and hair development were affected by genetic knockout of lamin B1 in keratinocytes ^8^. Moreover, mouse embryonic stem cells do not require any lamins for self-renewal and pluripotency ^9^.

Lamin B1 was found to be down regulated in bronchogenic carcinoma, colorectal cancer, and gastric cancer. On the contrary lamin B1 was elevated in prostate cancer ^10^. Thus, the lamin B1 role in both physiology and cancer biology are vague ^11^.

The present study aimed to determine the diagnostic and prognostic role of plasma levels of mRNA of lamin B1 by RT-qPCR as an early marker of HCC, in comparison to the traditional parameters of α-fetoprotein (AFP) levels, and ultrasound and triphasic computed tomography (CT) imaging. The study also aimed to reveal the correlation between lamin B1 mRNA levels with the tumor size and the number of tumor nodules, represented using the Barcelona-Clinic Liver Cancer (BCLC) Staging System ^12^, as well as to serum AFP level.

## Methods

### Subjects

This prospective cross-sectional pilot study was approved by the Research and Ethics Committee of Ain Shams University, Cairo, Egypt. Each patient provided verbal consent for the inclusion of their data in this study. Verbal consent was obtained over written consent, since some patients were illiterate (the ethical committee approved this form of consent).

This study was conducted at the Clinical Pathology Department in collaboration with the Department of Tropical Medicine and Infectious Diseases at Ain Shams University Hospitals from May 2016 to January 2017. The patients were consecutively recruited from the Tropical Medicine Department at Ain Shams University Hospitals and HCC clinic. The patients included 30 patients with primary HCC on top of viral cirrhosis (all were HCV positive). There were 27 males and 3 females (age range, 47–69 years), in addition to ten 10 cases with chronic liver diseases (7 males and 3 females; age range, 30–70 years) as patient controls and 10 healthy subjects age- and sex-matched subjects (7 males and 3 females; age range, 31–60 years), serving as healthy controls. HCC patients were classified into 3 stages using the BCLC Staging System: very early (Stage 0; n = 14), who had a single tumor <2 cm with child class A (according to the Child-Pugh system; 13) and performance status 0; early (Stage A; n=7), who had single or maximum 3 tumor nodules < 3 cm with child class A–B and performance status 0; and intermediate (Stage B; n=9), who had multinodular tumor child class A–B and performance status 0.

The diagnosis of HCC was based on non-invasive imaging techniques; either triphasic multidetector CT scan or dynamic contrast-enhanced magnetic resonance imaging, according to AASLD guidelines ^14^, and were only performed for cirrhotic patients. For patients with hepatic nodules beyond 1 cm in diameter, one imaging technique was required, while in those patients with smaller lesions, both techniques were performed for confirmation. Pathological diagnosis was performed for selected lesions, in which imaging studies did not demonstrate the typical HCC criteria.

Subjects with malignancies other than HCC, autoimmune diseases, chronic liver diseases other than viral hepatitis, benign liver tumors or secondary (metastatic) liver tumors and BCLC stage C or D disease were excluded from the study.

All individuals included in this study were subjected to a full assessment of medical history, focusing on previous hepatic disorders or predisposing factors preceding liver disease, thorough clinical examination, with special emphasis on abdominal examination, jaundice, edema and ascites, radiological investigations (including CT scan (for HCC patients only), abdominal ultrasound for patients with hepatic disorders and normal controls) Serum AFP was assayed by chemiluminescent-immunometric technique and plasma mRNA lamin B1 quantitated by RT-qPCR.

### Samples

A total of 4 ml venous blood was withdrawn from each subject; 2 ml were collected in EDTA K3 vacutainers for the lamin B1 assay and centrifuged at 1500 *g* for 10 minutes. Plasma was collected, aliquoted and stored at −70°C. The remaining 2 ml were collected in sterile vacutainers with a Z Serum Sep Clot Activator (Greiner Bio-One). Afterwards, blood was centrifuged for 10 min at 1000 *g*, the serum was used for immediate analysis of AFP.

### Assay of AFP

AFP was assayed by electro-chemiluminescence on a Cobas e411 immunoassay autoanalyzer (Roche Diagnostic Gmbh), using the AFP α1-fetoprotein immunoassay kit also provided by Roche.

### Assay of lamin B1 mRNA by RT-qPCR

RT-qPCR was performed through several steps, as follows. (i) RNA extraction from the EDTA-K3 plasma samples was performed using a ready-made extraction kit (miRNeasy Mini Kit) supplied by Qiagen, Inc. (ii) Real-time RT and cDNA synthesis was conducted using the extracted RNA with the QuantiTest reverse transcription kit (Qiagen, Inc.). Using these reagents, only mRNAs with 3'-poly (A) tails are templates for cDNA synthesis. cDNA synthesized with this system can be used as a template in the PCR reaction. (iii) DNA was amplified and detected by qPCR, using RT-PCR Master Mix kit supplied by Qiagen, Inc. Amplification was performed using the real-time light cycler Stratagene Mx3005P (Agilent Technologies, Inc.). qPCR was performed according to the following protocol: 5 min at 95°C (PCR initial activation step), followed by 45 cycles of 30 s at 95°C (two-step denaturation) and 30 s at 60°C (combined annealing and extension step with fluorescence data collection). A negative control containing all reagents except template RNA was included in each run. (iv) Results were reported in relative quantification, where the normalized level of target gene expression was calculated by using the 2 ^–ΔΔCq^ formula ^15^.

### Statistical analysis

Statistical analysis was performed using SPSS (version 22.0, IBM Corp.). Qualitative data were expressed as percentages, whereas quantitative data were expressed as mean ± standard deviation. Skewed data were expressed as median and inter-quartile range. The Kruskal–Wallis test (H test) was applied for statistical comparison between three or more sets of data if one or more of them had a skewed distribution. The Mann-Whitney U-test (Wilcoxon rank-sum test) was used to compare two independent sets of data if one or both of them had a skewed distribution. Spearman's rank correlation coefficient (r _s_) was used to assess the degree of correlation between two sets of variables if one or both of them showed a skewed distribution. The diagnostic performance of lamin B1 was evaluated in terms of its diagnostic sensitivity, specificity and efficacy. The area under the curve (AUC) was used to describe the overall test performance. Multi–ROC curve was applied to allow the comparison of different rules over varying test thresholds.

## Results

### Comparison between study groups

A highly statistically significant difference was found among the three studied groups with regards to both AFP and lamin B1 levels (H=23.4 and H=29.9, respectively; both p<0.01). Plasma levels of lamin B1 mRNA (2 ^-ΔΔCq^) was significantly higher in HCC patients than in chronic liver disease (CLD) patients and healthy controls with median (Q1–Q3) 3.9 (2–13.3), 0.9 (0.8–1.1) and 1 (0.9–1.2), respectively

Upon comparison between two groups individually, AFP was significantly higher in group I versus II and III (Z=3.4, p<0.01 and Z=4.1, p<0.001, respectively). Similarly, the median levels of lamin B1 was significantly higher in group I versus II & III (Z=4.3, p<0.001 and Z=4.3, p<0.001, respectively). There was no significant difference between group II and group III as regards AFP and lamin B1 (Z=1.4 and Z=0.9, p>0.05, respectively) ( Table 1).

**Table 1.** Comparison of serum AFP and lamin B1 between groups using Wilcoxson’s rank-sum test.

Parameter	Group I vs Group II		Group I vs Group III		Group II vs Group III	
	Z	p-value	Z	p-value	Z	p-value
AFP (ng/dl)	3.4	<0.01	4.1	<0.001	1.4	>0.05
Lamin B1 (2 ^−ΔΔCq^)	4.3	<0.001	4.3	<0.001	0.9	>0.05

AFP, α-fetoprotein

### Comparison between HCC stages

The median levels of AFP were 7.9 ng/dl in patients with stage 0 disease, 243 ng/dl in those with stage A disease and 251 ng/dl in those with stage B disease. Regarding lamin B1, median expression levels were lowest in patients with stage 0 disease ^2^, higher in those with stage A disease (5.1) and highest in stage B (19.4).

Comparison between the three stages of HCC patients showed a statistical significant difference in AFP ((H=9.9, p<0.01) and lamin B1 (H=24.4, p<0.001,). When comparing two stages individually, AFP was significantly lower in stage 0 vs stage A (p<0.05), in stage A vs stage B (p<0.05), and in stage 0 vs B (p<0.05). This statistical decrease was highly significant in case of lamin B1 (p<0.001, p<0.01, p<0.001 for stage 0 vs stage A, stage A vs stage B, and stage 0 vs B, respectively) ( Table 2).

**Table 2.** Comparison of AFP and lamin B1 between each two groups of the three stages HCC using Wilcoxson’s rank-sum test.

Parameter	Stage 0 vs Stage A		Stage 0 vs Stage B		Stage A vs Stage B	
	Z	p-value	Z	p-value	Z	p-value
AFP (ng/dl)	2.8	<0.05	2.4	<0.05	2.5	<0.05
Lamin B1 (2 ^−ΔΔCq^)	3.5	<0.001	3.9	<0.001	3.3	<0.01

AFP, α-fetoprotein.

### Comparison of study groups with different HCC stages

Comparison of group II with the three stages of HCC (0, A& B) revealed a significantly lower values in group II versus each of stage 0, A and B for both AFP (Z=2.1, Z=3.4 and Z=3.2; p<0.05, p<0.01 and p<0.01), respectively) and lamin B1 (Z=3.4, Z=3.4 and Z=3.7; p<0.01, p<0.01 and p<0.001, respectively) ( Table 3).

**Table 3.** Statistical comparisons between serum AFP and lamin B1 in Group II versus the three stages of HCC using Wilcoxson’s rank-sum test.

Parameter	Group II vs Stage 0		Group II vs Stage A		Group II vs Stage B	
	Z	p-value	Z	p-value	Z	p-value
AFP (ng/dl)	2.1	<0.05	3.4	<0.01	3.2	<0.01
Lamin B1 (2 ^−ΔΔCq^)	3.4	<0.01	3.4	<0.01	3.7	<0.001

AFP, α-fetoprotein

Regarding the tumor size and number of nodules, comparison between HCC patients with one tumor nodule (T1) and those with two or more tumor nodules (T2/Tm) revealed that the first group was statistically significantly lower than the second group for AFP (Z=3.1, p<0.01) and lamin B1 (Z=4.5, p<0.001) levels. Moreover, comparison between HCC patients with tumor size >3 cm revealed a significantly higher values versus those with tumor size ≤3 cm as regards AFP and lamin B1 (Z=3.1, p<0.01 and Z=4.6, p<0.001), respectively.

A significant positive correlation was observed between AFP and lamin B1 in group I (r=0.46, p<0.05), but not in group II or III (r=−0.11, p>0.05 and r=−0.41, p>0.05, respectively).

### Diagnostic accuracy of AFP and lamin B1

Table 4 shows receiver operating characteristic (ROC) curve analysis applied to the study results to examine the diagnostic performance of lamin B1 and AFP as tumor markers in HCC at different cut-off values. At a cut-off level of 5.0 ng/dl, the diagnostic performance of AFP for differentiation between HCC cases and the two control groups showed 80% sensitivity, 90% specificity, 75% negative predictive value (NPV), 92.3% positive predictive value (PPV) and 84% efficacy, with an area under the curve (AUC) =0.822. The best cut-off to differentiate between group II and stage 0 was 3.5 ng/dl, with a 78.6% sensitivity, 60% specificity, 73.3% PPV, 66.7% NPV, 70.8% efficacy and AUC =0.762; the cut-off of 142 ng/dl AFP was used to differentiate between stage A and stage B, with 71.4% sensitivity, 92.9% specificity, 83.3% PPV, 86.7% NPV, 85.7% efficacy and AUC =0.844.

**Table 4.** Diagnostic performance of AFP & Lamin B1 among different studied groups.

Parameter	Group/stage	Best cut-off	SN%	SP%	PPV%	NPV%	Eff%
AFP (ng/dl)	Group I vs II/III	5.0	80	90	92.3	75	84
	Stage 0 vs Group II	3.5	78.6	60	73.3	66.7	70.8
	Stage 0 vs group A	142	71.4	92.9	83.3	86.7	85.7
Lamin B1 (2 ^−ΔΔCq^)	Group I vs II/III	1.4	100	90	93.4	100	96
	Stage 0 vs Group II	1.3	100	90	93.3	100	95.8
	Stage 0 vs A	2.8	100	92.9	87.5	100	95.2
AFP (ng/dl) and Lamin B1 (2 ^−ΔΔCq^)	Group I vs II	3.5(ng/dl) + 1.4(2 ^−ΔΔCq^)	100	100	100	100	100

SN%, sensitivity; SP%, specificity; PPV%, positive predictive value; NPV%, negative predictive value; EFF%, efficacy; AFP, α-fetoprotein.

**Figure 1.**
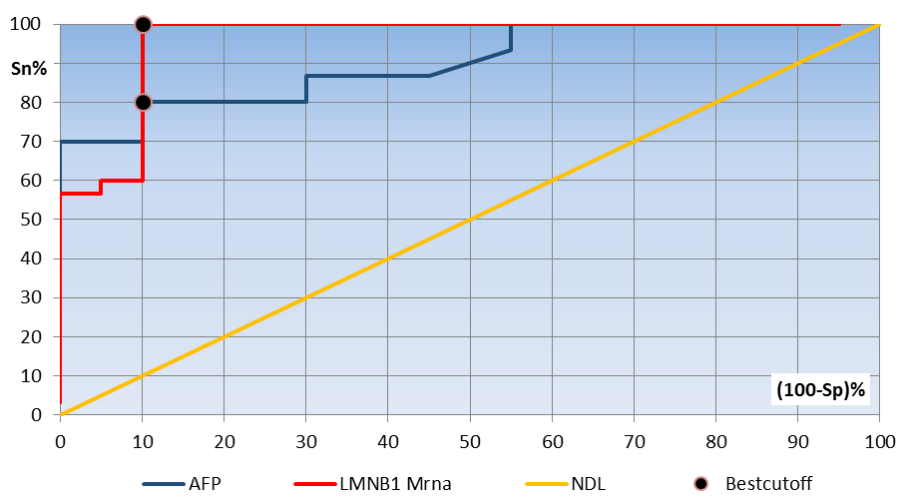
Receiver operating characteristics curve analysis showing the diagnostic performance of AFP and lamin B1 mRNA for discriminating patients with Group I from Group II and III. Area under the curve: AFP, 0.822; LMNB1 mRNA, 0.962.

**Figure 2.**
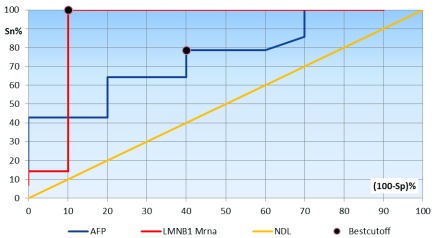
Receiver operating characteristics curve analysis showing the diagnostic performance of AFP and lamin B1 mRNA for discriminating patients with HCC Stage 0 from Group II (those with chronic liver disease). Area under the curve: AFP, 0.762; LMNB1 mRNA, 0.962.

Plasma lamin B1 mRNA showed a much better performance to differentiate between HCC cases and the two control groups, where, at a 2 ^–ΔΔCq^ cut-off of 1.4, sensitivity was 100%, specificity was 90%, NPV was 100%, PPV was 93.4% and efficacy was 96%, with an AUC =0.962. A 2 ^–ΔΔCq^ cut-off of 1.3 was used to differentiate between patients with stage 0 HCC and CLD, with 100% sensitivity, 90% specificity, 100% NPV, 93.3% PPV, 95.8% efficacy and AUC =0.926. A 2 ^–ΔΔCq^ cut-off of 2.8 was used to differentiate between patients with stage 0 and A, yielding 100% sensitivity, 92.9% specificity, 100% NPV, 87.5% PPV, 95.2% efficacy and AUC 0.972 ( Figure 1 and Figure 2).

**Figure 3.**
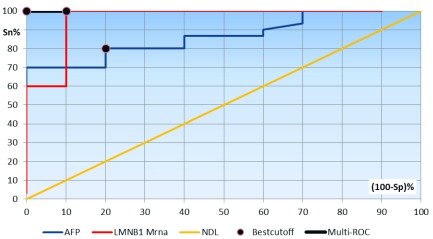
Multi-receiver operating characteristics curve analysis showing the diagnostic performance of both AFP and lamin B1 mRNA and their combination for discriminating patients with HCC from chronic liver disease. Area under the curve: AFP, 0.844; LMNB1 mRNA, 0.957; multi-ROC, 1.000.

Multi-ROC curve analysis was constructed to assess the diagnostic performance of a combination of both AFP (at a cut-off value of 3.5 ng/dl) and lamin B1 (at a 2 ^–ΔΔCq^ cut-off value of 1.4) to discriminate between patients with HCC and those with CLD. At these cut-off values, the diagnostic sensitivity was 100%, specificity 100%, PPV 100%, NPV 100% and efficacy 100% ( Figure 3).

Complete raw data associated with the study, including demographic information, infection status, tumor characteristics and Cq values Click here for additional data file. Copyright: © 2018 Abdelghany AM et al. 2018 Data associated with the article are available under the terms of the Creative Commons Zero "No rights reserved" data waiver (CC0 1.0 Public domain dedication).

## Discussion

Results of the present study revealed that there was a significantly higher level of AFP in group I (median =31.6 ng/dl) compared to chronic liver diseases patients (median= 3.5 ng/dl) and healthy controls group (median= 1.3 ng/dl). This was in agreement with the findings of Wei *et al.* ^16^, who proved that AFP levels are significantly higher in patients with HCC in comparison to those with chronic liver disease (CLD) and healthy controls; they suggested that this increase is due to selective transcriptional activation of the AFP gene in the malignant hepatocytes.

Plasma levels of lamin B1 mRNA (2 ^-ΔΔCq^) was significantly higher in HCC patients than in CLD patients and healthy controls. Similar results were achieved by Wong and Luk ^17^. Sun *et al.* ^18^ found that lamin B1 mRNA was detected in the plasma of 82% of group 1 subjects, whereas it was detected only in 19% of group 2 subjects and in 17% of those in group 3. Levels of lamin B1 mRNA were also significantly upregulated in patients with HCC.

The accumulated lamin B1 is released from the HCC cells, which have altered metabolism and are in state of oxidative stress, stimulating the p38 MAPK pathway. In addition, autoantibodies against lamin B1 were found to be positive in 17% of HCC patients, but none was found in CLD and healthy controls ^18^. This may be attributed to circulating lamin B1 mRNA in plasma that results from lysis of cancer cells or being encoded by tumor-related genes ^19^.

In the present study, statistical comparison of different Barcelona Stages in patients with HCC revealed that AFP levels were significantly higher in stages A and B (median, 243 and 251 ng/dl, respectively) than in stage 0 (median, 7.9 ng/dl). These results are in agreement with those of Peng *et al.* ^20^, who found a significant increase in AFP levels corresponding to Barcelona Staging. This is also in accordance with the results of Zhang *et al.* ^21^, who concluded that AFP can act as an independent prognostic factor for HCC, as it can induce the malignant progression of liver cancer via tumorigenesis and cellular growth, migration and invasion.

With regards to plasma levels of lamin B1 (2 ^-ΔΔCq^), there was a significant increase in patients with stages A and B HCC compared to patients with stage 0 disease, with a median (Q1–Q3) in stage 0, A and B of 2.0 (1.7–2.4), 5.1 (3.7–6) and 19.1 (14.7–28.2), respectively. Similarly, *Sun et al.* ^18^, using western blot analysis, found that the expression of lamin B1 was positive in 71% of patients with early-stage HCC and positive in 83% in patients with late-stage HCC. This suggests that lamin B1 could induce increased invasiveness and promote progression. This may be related to the fact that lamin B1 is present in actively developing tumors ^17^.

 The potential value of lamin B1 as an early diagnostic marker of HCC was demonstrated in the present study, where a statistically significant increase in plasma level among stage 0 HCC patients was observed compared to CLD patients (group II). This indicates its importance in detecting the very early cases of HCC. In accordance with the present results, Sun *et al.* ^18^ demonstrated that lamin B1 mRNA plasma levels were elevated in 76% of early cases of HCC, compared to 19% only in cirrhotic patients. The same sensitivity was revealed by Wong and Luk ^17^. Lim *et al.* ^22^ found that the expression level of the protein in cirrhotic tissue samples increased and rose even more in the tumorous tissue samples using MALDI-TOF mass spectrometry. This is due to the specific involvement of lamin B1 in carcinogenesis, since it increases in cancer cells more than cirrhotic cells ^17^.

Comparison between patients with stage 0 HCC versus those with stages A and B disease revealed that the AFP levels in those with stage 0 disease was statistically significantly lower than in those with stages A and B. Similarly, AFP levels in patients with HCC with a tumor size >3 cm were statistically different to those in patients with a tumor size ≤3 cm. This is in agreement with the results of Peng *et al.* ^20^, who revealed that serum AFP correlated with tumor size and high AFP (>200 ng/dl) was associated with large tumors (>5 cm).

Previous studies have revealed that serum AFP levels was found to be increased in HCC patients, with these increased levels being positively associated with tumor size and number of tumors ^24, 25^. This finding was in agreement with the postulation that AFP can act as a growth regulator. Increased proliferation *in vitro* in response to AFP has been observed for developing or embryonic cells and human hepatoma cells, but not untransformed cells, owing to the absence of specific membrane AFP receptors ^26^.

In an attempt to study the prognostic significance of lamin B1, lamin B1 mRNA plasma levels (2 ^-ΔΔCq^) was compared between patients with one tumor nodule and those with two or more nodules. A statistically significant difference was demonstrated, with median (Q1–Q3), 2.0 (1.7–2.4) and 10.95 (5.1–22.3), respectively. Similarly there was a significant difference in lamin B1 expression in patients with tumor sizes ≤3 cm versus those with tumors >3 cm, with median (Q1–Q3) values of 2.0 (1.7–2.4) and 10.9 (5.1–22.3), respectively.

Similar results were achieved by Sun *et al.* ^18^, who used proteomic analysis to demonstrate that overexpression of lamin B1 was significantly associated with an increased number of tumor nodules and the size of tumors. On applying conventional RT-PCR, (not real time PCR) there was an increase in the positivity rate of circulating lamin B1 mRNA that gradually increased with tumor stage progression. This could be related to the phosphorylation of lamin B1 mediated by phospholipase C1, resulting in cell proliferation via G _2_/M cell cycle progression, eventually increasing the tumor size and number ^26^.

 In the current study, there was a positive correlation between AFP and lamin B levels, in HCC patients. However, this disagreed with the results of Sun *et al.* ^18^, who found no correlation between the two markers. This might be due to different techniques and different studied populations in the two studies.

The diagnostic performance of AFP was assessed using ROC curve analysis. At a cut-off level of 5.0 ng/dl, AFP was able to differentiate between HCC cases and the 2 control groups with 80% sensitivity, 90% specificity, 75% NPV, 92.3% PPV and 84% efficacy. These results were comparable to those of Kim *et al*. ^26^, who, at a cut-off 70.4 ng/dl, had a rather lower diagnostic sensitivity (54.8%) but a higher specificity (100%). In a study by Yang *et al.* ^27^, AFP in HCC patients revealed lower diagnostic sensitivity and specificity (of 68.7% and 61.9%, respectively).

On the other hand, plasma lamin B1 mRNA showed much better performance in differentiation between group 1 and groups 2 and 3, with 100% sensitivity, 90% specificity, 100% NPV, 93.4% PPV and 96% efficacy. Sun *et al.* ^18^, observed a comparable diagnostic performance, with a sensitivity of 86% and specificity of 80%. Similarly, Wong and Luk ^17^, showed a lower sensitivity (76%) and specificity (82%) for the detection of HCC when assessed with cirrhotic patients and healthy controls. Moreover, Liu *et al.* ^19^, revealed that lamin B1 had a diagnostic performance of 100% sensitivity, which was similar to the current study, but a much lower specificity of 27%, 100% PPV and 70% NPV.

In conclusion, measurement of lamin B1 mRNA is highly recommended in patients with CLD with normal serum AFP, especially in known cirrhotic patients that deteriorate rapidly without any apparent etiology. Addition of plasma lamin B1 mRNA to the current standard tests for diagnosis of HCC as a new diagnostic and screening tool could greatly improve the ability to identify such patients and thus could allow them to benefit from earlier treatment.

## Data availability

The data referenced by this article are under copyright with the following copyright statement: Copyright: © 2018 Abdelghany AM et al.

Data associated with the article are available under the terms of the Creative Commons Zero "No rights reserved" data waiver (CC0 1.0 Public domain dedication). 

**Dataset 1. Complete raw data associated with the study, including demographic information, infection status, tumor characteristics and Cq values.** DOI: https://doi.org/10.5256/f1000research.14795.d212413 ^28^.
