# Supplementary material for: Using Lamin B1 mRNA for the early diagnosis of hepatocellular carcinoma: a cross-sectional diagnostic accuracy study
Source: F1000Res. 2018 Aug 24;7:1339. [Version 1] doi: 10.12688/f1000research.14795.1 (PMC6208568; doi:10.12688/f1000research.14795.1)
Supplement: Peer review history

# Peer review: Referee response for version 1

**DOI:** 10.5256/f1000research.16100.r39775

**Published:** 2018-10-30

**Authors:** Sherief Abd-Elsalam

**Affiliations:** 1 Department of Tropical Medicine and Infectious Diseases, Faculty of Medicine, Tanta University, Tanta, Egypt

The authors investigated Lamin B1 mRNA for the early diagnosis of hepatocellular carcinoma. A good job you did. The work is technically sound and the work is clearly presented. However, it needs adding important recent references about prevalence of HCC in Egypt. Additionally, there are minor comments need to be addressed:

 In introduction; the data about HCC prevalence in egypt is too old since 2003; is too old; you should add updated citations about HCC prevalence in Egypt; I suggest these two references:

- Prevalence of hepatocellular carcinoma in chronic hepatitis C patients in Mid Delta, Egypt: A single center study ^1^. 
- Epidemiology of liver cancer in Nile delta over a decade: A single-center study ^2^. 

​​​​In methods; you should clarify why the small number of patients in group II, III. Is it the cost?

 In discussion, You should add limitations of the study. The most important one is small sample size.

I have read this submission. I believe that I have an appropriate level of expertise to confirm that it is of an acceptable scientific standard.

## References

[1] ZiadaDHEl SadanySSolimanHAbd-ElsalamSSalamaMHawashNSelimAHamisaMElsabaghHM: Prevalence of hepatocellular carcinoma in chronic hepatitis C patients in Mid Delta, Egypt: A single center study. *J Egypt Natl Canc Inst*.2016;28(4) : 10.1016/j.jnci.2016.06.001 257-262 10.1016/j.jnci.2016.06.001 27378258

[2] Abd-ElsalamSElwanNSolimanHZiadaDElkhalawanyWSalamaMHawashNArafaMBadawiRShehataWMKhalilHSElmashadN: Epidemiology of liver cancer in Nile delta over a decade: A single-center study. *South Asian J Cancer*.7(1) : 10.4103/sajc.sajc_82_17 24-26 10.4103/sajc.sajc_82_17 29600229PMC5865090

# Peer review: Referee response for version 1

**DOI:** 10.5256/f1000research.16100.r37615

**Published:** 2018-09-06

**Authors:** Dina Fekry

**Affiliations:** 1 Department of Clinical Pathology, National Research Centre (NRC), Cairo, Egypt

HCC is the most common primary malignancy of hepatocytes.It is one of the most common causes of cancer world wide.

 The nuclear Lamina is a proteinaceous meshwork and it is essential for a number of cellular functions. Lamin B1 was found to be essential for nuclear integrity and cell survival.

 This article aimed at determination of the diagnostic as well as prognostic value of Lamin B1 in HCC patients together with AFP.

 The study was carried out on 30 patients with HCC which were further classified into stage 0,A and B according to size and number of tumor nodules,10 patients with CLD considered as patient controls together with 10 healthy patients considered as healthy controls.

 Measurement of mRNA of Lamin B1 was done by RT-qPCR however, measurement of AFP was done by electrochemiluminescent assay.

 Statistical analysis was done using SPSS( version 22.0, IBM corp.).MultiROC curve was used to evaluate the diagnostic sensitivity and specificity of Lamin B1in HCC.

 Presented results showed that AFP was significantly higher ion HCC patients when compared to other groups.

 As for Lamin B1, it was significantly higher in stages A and B in HCC patients when compared to 0 stage. Besides, it was significantly higher in stage 0 compared to CLD giving it a prevalage as an early marker of HCC. Also, its prognostic significance was clear since it was significantly higher in tumors with bigger nodules.

 In my opinion, the article showed full information of methods and analysis however, authors may consider to include the Barcelona classification of HCC in the abstract in order to understand the results which include 0, A, B stages.

 In addition, mentioning the relation of Lamin B1 to liver cell and how it affects liver cell integrity may be of great importance for the core of the article.

 The statistical analysis was impressing and totally appropriate however, correlation between AFP and Lamin B1 in HCC group would be preferably introduced in a table.

 Finally, the conclusions drawn were adequately supported by the presented results.

I have read this submission. I believe that I have an appropriate level of expertise to confirm that it is of an acceptable scientific standard.
